# A narrative review on radiographic progression to monitor psoriatic arthritis activity

**DOI:** 10.1186/s42358-026-00557-9

**Published:** 2026-06-17

**Authors:** Carlos García-Porrúa, José Antonio Pinto Tasende, Evelin Cecilia Cervantes Pérez, Rafael Benito Melero-González, Francisco José Maceiras-Pan

**Affiliations:** 1 Rheumatology Department, Hospital Universitario Lucus Augusti, Avda. Dr. Ulises Romero 1, Lugo, 27003 Spain; 2 Division of Rheumatology, INIBIC, Complexo Hospitalario Universitario de A Coruña, 84 Xubias de Arriba St, A Coruña, 15009 Spain; 3 Rheumatology Department, Complexo Hospitalario Universitario de Pontevedra, Dr. Loureiro Crespo 2, Pontevedra, 36001 Spain; 4 Rheumatology Department, Complexo Hospitalario Universitario de Ourense, Calle Ramon Puga Noguerol, 54, Ourense, 32005 Spain; 5 Rheumatology Department, Complejo Hospitalario Universitario de Vigo, Vigo, Spain

**Keywords:** Biologic, Disease-modifying antirheumatic drugs (DMARDs), Cytokines, Psoriatic arthritis, Radiographic progression

## Abstract

Psoriatic arthritis (PsA) is a systemic chronic inflammatory disease characterized by arthritis and structural damage of joints associated with persistent inflammation. Radiographic progression (RP) is typically used to measure the development of structural damage using different semiquantitative scoring methods. An important therapeutic goal is to prevent structural damage. Visualizing such damage using RP seems to predict shorter survival in patients with PsA. Therapeutic agents that inhibit structural damage are considered disease-modifying in PsA. There are no validated and clinically useful biomarkers for stratifying patients and informing clinical treatment decisions to increase the likelihood of a response to any given therapy. This narrative review examines current approaches for assessing the extent of structural damage in PsA, monitoring of PsA disease activity, risk factors that contribute to the progression of RP in PsA, and discusses the efficacy (inhibition of RP) of the new approved therapies that have emerged over the last few years for use in PsA. While there are unmet needs to clarify and define RP, the extent of structural damage in the peripheral forms of PsA was most frequently determined using the PsA-modified Sharp and Sharp-van der Heijde Rheumatoid Arthritis scoring methods. Factors that lead to a more aggressive, faster, or more active RP are also related to the number of activity indicators (overweight, smoking, etc.). In patients with high psoriatic activity and thus greater disease progression, determining structural damage at 6 months of follow-up may be sufficiently sensitive to obtain RP information and evaluate the evolution of the disease.

## Introduction

Psoriatic arthritis (PsA) is a systemic chronic inflammatory arthritis that is found in approximately 30% of individuals with the skin condition, psoriasis [1, 2]. PsA is characterized by a range of musculoskeletal and extra-articular disease manifestations (e.g. dactylitis, enthesitis, peripheral arthritis, psoriasis of the skin and nails, spondylitis) and persistent inflammation. This ongoing inflammation can damage cartilage and bone, causing progressive joint damage that can lead to functional impairment and disability, accompanied by a substantial reduction in quality of life [1, 3–5].

Several challenges or unmet needs surround the diagnosis and management of PsA, including the lack of an algorithm for early diagnosis, the lack of clinical/molecular risk factors for PsA in individuals with cutaneous psoriasis and the inability to predict which patients with PsA are likely to experience joint damage. Furthermore, there are no validated and clinically useful biomarkers to help stratify patients and inform clinical treatment decisions to increase the likelihood of a response to any given therapy [2]. Evidence of radiographic change is a prognostic factor for shorter survival in patients with PsA, so inhibiting the progression of structural damage is an important therapeutic goal, and a characteristic of disease-modifying therapies in PsA [6]. Various semiquantitative methods for scoring radiographic progression (RP) that have been developed to assess structural damage in PsA have proven very reliable and important in clinical research, and have been used to identify effective therapies for patients with PsA [6]. However, none of these scoring methods has demonstrated adequate feasibility and sensitivity for application in large, longitudinal studies, and they may have limited application in the clinical setting [6]. Furthermore, when employing tools to assess the pathology of peripheral (hands, wrists and/or feet) and axial (spinal and/or sacroiliac) joints, Antony and colleagues found that no specific instruments provided evidence for thresholds of meaning or for use in clinical trials [7].

Multiple factors contribute to the pathogenesis of PsA, including genetic predisposition, sex differences, environmental triggers, and immunological dysfunction [1]. Disease activity indices measure the extent of RP in PsA, with higher disease activity scores indicating a greater extent of RP, emphasizing the importance of suppressing inflammation as a main objective of patient management [8, 9]. New approved therapies for use in PsA that have emerged over the last few years include targeted therapeutic agents capable of stabilizing bone turnover and inhibiting radiographic joint damage, and potentially able to prevent generalized bone loss [10].

This narrative review examines current approaches for assessing the extent of structural damage in PsA, and how PsA disease activity is monitored in the clinic. The review also considers what risk factors contribute to the progression of RP in PsA, and appraises the efficacy regarding inhibition of RP of the new approved therapies that have emerged over the last few years for use in PsA, with the aim of developing recommendations for rheumatologists in clinical practice.

## Methods

### Literature search and data selection

Research articles were identified for this narrative review through a search on 04 April 2024 of the PubMed database using the following search terms: ‘psoriatic arthritis’ in combination with (using the Boolean operator “OR”) the MeSH terms ‘radiographic progression’, ‘radiographic damage’, and ‘structural damage’, and data related to the following therapies: anti-tumor necrosis factor (anti-TNF) agents infliximab, etanercept, adalimumab, certolizumab, and golimumab; the anti-interleukin (IL)-12/23 agent ustekinumab; anti-IL-23 agents risankizumab, and guselkumab; anti-IL-17A agents secukinumab, and ixekizumab; the anti-IL-17A/F antibody bimekizumab; the Janus kinase (JAK) inhibitors tofacitinib, and upadacitinib; the phosphodiesterase-4 (PDE4) inhibitor apremilast; and the selective co-stimulation modulator abatacept.

All English-language articles were identified up to 04 April 2024, with no initial data limit set. A total of 516 records were identified. Duplicates were removed, and records filtered to remove those involving in vitro or preclinical data, as well as reviews that have been superseded with more updated material. Of the remaining records, the authors identified relevant papers for inclusion in this narrative review. Other content for this article was identified by a manual search of reference lists in relevant papers, as well as the authors’ experience in this area.

## Results and discussion

### Radiographic determination of structural damage in PsA

Conventional radiographs are the standard approach for assessing the extent of structural damage in PsA, with almost half of patients exhibiting structural damage and functional impairment within 2 years of symptom onset [6]. Radiographs reveal the presence of periostitis (inflammation of the periosteum surrounding the bones), joint damage (such as ankylosis, bone erosions, osteolysis, and subluxation), and any involvement of the sacroiliac and spinal joints, and identify bone spurs [6]. Several semiquantitative radiographic scoring systems have been developed to assess severity and progression over time of peripheral joints in patients with PsA. These include the Steinbrocker global scoring method for classifying functional and radiographic damage in rheumatoid arthritis (RA) modified for use in PsA [11–13], the PsA-modified Sharp and Sharp-van der Heijde (SvdH) RA scoring methods [14], and the PsA Ratingen score (PARS) [14, 15] (Table 1). Higher scores on these scoring systems reflect findings of increasingly severe structural damage such as erosions, joint space narrowing, and ankylosis [9].

**Table 1:** Current scoring methods used to measure radiographic progression in psoriatic arthritis, with randomized clinical trial evidence supporting the utility of radiographic assessment of structural damage as an outcome in psoriatic arthritis

Scoring method	Time to final radiographic assessment, week	Clinical Trial (study treatment)
Typical modified Sharp used for PsA	24, 48	Phase 3 study (etanercept vs. placebo) [16, 17] ADEPT (adalimumab vs. placebo) [18, 19]
Sharp van der Heijde for PsA	12, 16, 24, 48, 50, 52, 54	IMPACT (infliximab vs. placebo) [20] IMPACT 2 (infliximab vs. placebo) [21] GO-REVEAL (golimumab vs. placebo) [22] GO-VIBRANT (golimumab vs. placebo) [23] RAPID-PsA (certolizumab pegol) [24, 25] PSUMMIT-1 and − 2 (ustekinumab vs. placebo) [26] FUTURE-1 (secukinumab vs. placebo) [27] FUTURE-5 (secukinumab vs. placebo) [28] SPIRIT-P1 (ixekizumab vs. placebo vs. adalimumab) [29] ASTRACEA (abatacept vs. placebo) [30] OPAL Broaden (tofacitinib vs. adalimumab vs. placebo) [31, 32] SEAM-PsA (methotrexate vs. etanercept vs. combination) [33]

ADEPT, Adalimumab Effectiveness in Psoriatic Arthritis Trial; ASTRAEA, Active PSoriaTic Arthritis RAndomizEd TriAl; GO-REVEAL, Golimumab – A Randomized Evaluation of Safety and Efficacy in Subjects with Psoriatic Arthritis Using a Human Anti-TNF Monoclonal Antibody; GO-VIBRANT, Golimumab, an Anti-TNFα Monoclonal Antibody, Administered Intravenously, in Subjects With Active Psoriatic Arthritis; IMPACT, Infliximab Multinational Psoriatic Arthritis Controlled Trial; OPAL, Oral Psoriatic Arthritis Trial; PsA, psoriatic arthritis; SEAM-PsA, Study of Etanercept and Methotrexate in Combination or as Monotherapy in Subjects with Psoriatic Arthritis

Randomized clinical trial data support the utility of radiographic assessment of structural damage as an outcome in PsA [6] (Table 1). The radiographic scoring methods used by the modified Steinbrocker method, the PsA-modified Sharp score, the SvdH method, and the PARS have demonstrated face validity, reasonable inter- and intra-observer reliability, and sensitivity to change [12]. All methods are feasible for assessing RP in the peripheral joints of patients with PsA in clinical trials, with the SvdH method for PsA in particular showing good sensitivity and reliability for evaluating treatment in studies of biological agents [6].

International treat-to-target (T2T) recommendations on the management of axial spondyloarthritis (axSpA, which includes PsA) have been issued by the Assessment of SpondyloArthritis international Society–European League Against Rheumatism (ASAS-EULAR), and the 2019 international ASAS quality standard set for optimizing health and care services for patients with axSpA.

These recommendations advise regular monitoring of disease activity using validated outcome measures to assess the achievement of treatment targets [34–37]. In PsA clinical practice, T2T targets aim to achieve remission (inactive disease) or a state of low or minimal disease activity (MDA). Different disease activity measures are used as treatment targets, including the Psoriatic Arthritis Disease Activity Score (PASDAS), the Composite Psoriatic Disease Activity Index (CPDAI), the articular target Disease Activity index for PsA (DAPSA), the Group for Research and Assessment of Psoriasis and Psoriatic Arthritis Composite Exercise (GRACE), and MDA indices [8, 38–42]. The 2023 updated EULAR recommendations for the pharmacological management of PsA emphasize the abrogation of inflammation when treating patients with PsA, to prevent structural damage [37].

### Monitoring psoriatic arthritis disease activity in the clinic

The composite outcome measurements described above (e.g. the PASDAS, GRACE, and MDA criteria) capture disease activity across multiple domains, but are time-consuming and therefore challenging for routine clinical use [42]. DAPSA was found to be more sensitive to change in patients with distal interphalangeal joint involvement [38].

Interest has been expressed in the identification of biomarkers that could serve as substitutes for clinically meaningful endpoints, which would offer clinicians a standardized and objective way of monitoring patients, potentially simplifying head-to-head comparisons of treatments [42], and detecting structural damage. Although no specific diagnostic biomarker has yet demonstrated that it can detect joint involvement in patients with PsA [43], significantly higher serum IL-6 levels have been found in those with psoriasis and inflammatory joint disease in comparison to patients with skin disease only [44]. In addition, different inflammatory and proinflammatory biomarkers appear to be associated with different disease characteristics and phenotypes in PsA. Significant associations have been found between clinically active PsA disease and soluble levels of C-reactive protein (CRP), IL-6, IL-16, calprotectin, IL-12/IL-23p40, and intercellular adhesion molecule-1 (ICAM-1) compared with absent or low disease activity [45]. Different serological biomarkers may be associated with different PsA disease phenotypes, with higher levels of IL-6, IL-16, and macrophage inflammatory protein-1 beta (MIP-1β) in patients with axial disease (with or without concomitant peripheral disease) than in those without axial disease, higher levels of IL-6, serum amyloid A (SAA), CRP, and IL-8, compared with patients with mono-/oligoarthritic disease [45]. Elevated serum levels of calprotectin and S100A12 have been associated with peripheral radiographic features in patients with PsA [46], and positivity for anti-cyclic citrullinated peptide (CCP) antibodies has been associated with more radiographic damage and polyarticular involvement in patients with PsA [47].

### Risk factors predisposing to radiographic progression

Several risk factors such as PsA mutilans (PsAM), joint damage, radiological sex-specific differences, ethnic differences, axial involvement in psoriatic arthritis, clinical and biochemical inflammation, and tobacco smoking contribute to structural damage in PsA are described in detail below and summarized as top-level results in Table 2.

**Table 2:** A summary of literature detailing risk factors predisposing to radiographic progression in psoriatic arthritis

PsAM	• More radiographic axial disease/sacroiliitis was identified in radiographs of patients with PsAM versus radiographs of non-PsAM cases [37]
Joint damage	• The number of damaged joints was significantly correlated with radiological evidence of sacroiliitis [38] • Joint tenderness was positively associated with RP in PsA [39]
Radiological sex-specific differences	• In psoriatic SpA, women exhibited more aggressive peripheral disease than disease [40] • Axial involvement and radiographic peripheral joint damage more likely in men with PsA versus women with PsA [41] • Male sex a risk factor for early radiographic damage in PsA [42] • No sex-related differences in the degree of radiographic damage in axPsA [43] • A 2023 systematic literature review could not conclude that radiographic changes occur in axPsA [44]
Ethnic differences	• PsA radiographic phenotypes and disease activity differed between patients of South Asian origin versus those of North European origin [45]
Risk factors for axial involvement	• Radiographic damage to peripheral joints [46] • HLA-B27 positivity [47] • No association with HLA-B27 positivity [48]
Chemical and biochemical inflammation	• Higher swollen joint counts and elevated CRP [49] • Elevated baseline CRP was a strong independent risk factor for RP [50] • Elevated CRP at first rheumatologist visit linked to radiographic damage and PsA disease refractory to csDMARDs and TNF inhibitors [51] • PsA cases exhibited disordered systemic expression of soluble factors that promote osteoclastogenesis [52] • Serum calprotectin levels significantly associated with relapse in PsA [53] • No association between anemia and spinal radiographic progression in axSpA [54]
Gene expression profiling, DNA, and RP	• Erosive PsA linked to overexpression of genes involved in immunomodulatory processes and in PsA manifestations [55] • The G allele of IL-1B (-511 A/C) polymorphism linked to higher peripheral joint disease activity [56]
Tobacco smoking	• Linked to structural damage in PsA [57] • No association between tobacco smoking and structural damage in PsA [58]

axPsA, axial involvement in psoriatic arthritis; CRP, C-reactive protein; csDMARD, conventional synthetic disease-modifying antirheumatic drug; IL-1B, interleukin-1 beta; PsA, psoriatic arthritis; PsAM, psoriatic arthritis mutilans; RP, radiographic progression; SpA, spondyloarthritis; TNF, tumor necrosis factor

#### Psoriatic arthritis mutilans

A retrospective cohort study has recorded more radiographic axial disease/sacroiliitis in serial hand and feet radiographs of patients with PsAM than in radiographs of those with non-PsAM attending a teaching hospital; the development of PsAM did not appear to be prevented by use of conventional synthetic disease-modifying antirheumatic drugs (csDMARDs) or TNF inhibitors [48].

#### Joint damage

While peripheral joint damage predominantly characterizes RA, PsA affects both axial and peripheral joints with a mixed erosive-proliferative phenotype [49]. Ratingen and modified Sharp van der Heijde scores have shown some evidence for reliability, cross-sectional construct validity, and longitudinal construct validity as peripheral radiographic instruments to assess joint damage [7]. Conversely, evidence for the measurement properties of the instruments that evaluated axial joints was limited [7].

In a South African cohort of patients with PsA attending arthritis clinics, the number of damaged peripheral and/or axial joints was significantly correlated with radiological evidence of sacroiliitis, showing that prolonged inflammation leads to joint damage [50]. A positive but not significant association has been reported between hand joint tenderness and RP in patients with PsA, with the additional risk factors of erosion and power Doppler signs of synovitis showing a marked effect on subsequent structural damage [51].

#### Radiological sex-specific differences

Radiological sex-specific differences have been found in a cohort of patients with psoriatic spondyloarthropathy, with women exhibiting more aggressive peripheral disease (peripheral erosions and joint count) than men [52]. In that analysis, the HLA-B27 antigen was more prevalent among patients with isolated axial disease, most of whom were men [52]. Similarly, another study found that men with PsA were more likely to develop axial involvement and radiographic peripheral joint damage than women with PsA [53]. These findings are supported by 5-year follow-up data from the Swedish Early Psoriatic Arthritis Registry, showing that male sex was a risk factor for early radiographic damage and that achieving remission or MDA after 5 years was mainly protective against structural damage in men [54]. Conversely, no sex-related differences in the degree of radiographic damage or the frequency of HLA-B27 were found in a cohort of men and women with PsA axial disease [55]. Interestingly, although a 2023 systematic review of published literature found evidence for sex-specific differences in clinical characteristics, disease activity, and patient-reported outcomes in men and women with PsA, the review could not determine from the included studies whether radiographic changes occurred in patients with axial disease [56].

#### Ethnic differences

In a cross-sectional observational UK study that compared radiographic phenotypes in patients of South Asian origin and those of North European origin with a diagnosis of PsA, the South Asian patients had more tender and swollen peripheral joints, and worse disease activity as measured by the PASDAS, although SvdH scores did not differ between the two groups [57].

#### Risk factors for axial involvement in psoriatic arthritis

Radiographic damage to peripheral joints has been found to increase the risk for axial involvement in patients with PsA [58], and one study has shown that patients with axSpA and HLA-B27-positivity exhibit more severe radiographic damage than patients with axSpA who are negative for HLA-B27 [59]. Another study has reported finding no association between the presence of HLA-B27 and peripheral joint damage in patients with PsA [60].

#### Clinical and biochemical inflammation

Both clinical and biochemical inflammation (higher swollen peripheral and/or axial joint counts and elevated CRP) have been found to impact upon structural progression in patients with PsA [61]. The least amount of RP is observed in the absence of both clinical or biochemical inflammation, progression is higher in the presence of either clinical or biochemical inflammation, and highest when both are present [61]. A post-hoc sub-analysis of data from the randomized controlled trial Adalimumab Effectiveness in PsA Trial (ADEPT) assessed risk for RP from baseline to week 24 for CRP and other baseline variables [62]. The analysis found that elevated baseline CRP was a strong independent risk factor for RP, and that adalimumab treatment not only substantially reduced the overall risk of RP, but also provided the greatest radiographic benefit for patients with the highest baseline CRP values [62].

Long-term follow-up data from a cohort of patients with PsA have documented that presenting with elevated CRP at first visit to a rheumatologist was significantly associated with radiographic damage and disease that was significantly more refractory to both csDMARDs and TNF inhibitors [63]. A similar pattern was observed among those patients with repeatedly higher inflammatory markers during follow-up, while patients without elevations in CRP during follow-up had significantly milder disease with fewer erosions, less sacroiliitis and less need for TNF inhibitor therapy [63]. Some research has observed disordered systemic expression of soluble factors that promote osteoclastogenesis in patients with PsA compared with psoriasis and healthy controls [64]. This research has suggested that circulating mediators of bone remodeling may contribute to periarticular bone loss [64]. In a cohort of 56 patients with PsA receiving TNF inhibitors in remission or with low disease activity (28-joint Disease Activity Score [DAS28] ≤ 3.2 [DAS28 scores are on a scale from 1 to 10; lower scores reflect greater wellness]), serum calprotectin levels were significantly associated with disease relapse (defined as current DAS28 > 3.2 and an increase in DAS28 > 0.6 compared with baseline) during 12 months of follow-up [65]. Surprisingly, although anemia represents a biomarker for increased radiographic damage in RA, anemia has not been found to predict spinal radiographic progression in patients with axSpA [66].

#### Tobacco smoking

The effects of tobacco upon disease activity in PsA are controversial. Some literature reports that tobacco smoking is associated with structural damage in patients with PsA [67], whereas a later analysis of the available evidence has found no such association [68].

### Current therapies that impact upon radiographic progression

The approval of new pharmacological options targeting various modes of action for PsA (in addition to skin psoriasis) has extended the management options over the last few years. Licensed drugs for PsA now include csDMARDs, such as methotrexate (MTX), leflunomide, and sulfasalazine, biological DMARDs (bDMARDs) that target TNF, the IL-12/23 or IL-23 pathway, the IL-17 A and IL-17 A/F pathways, and the cytotoxic T-lymphocyte-associated antigen 4 (CTLA4) signaling molecule (Table 3). Targeted synthetic (ts) DMARDs that inhibit Janus kinases (JAKs) or phosphodiesterase 4 (PDE4) are also available (Table 3). Pivotal clinical trials and real-world evidence have assessed the impact of these approved treatments upon RP in PsA (Table 3) [16–33, 48, 50–89].

**Table 3:** Current pharmacological options approved for use in PsA (as at April 2014) with evidence from pivotal phase 3 trials or real-world data demonstrating treatment efficacy in preventing or inhibiting radiographic progression

Type of DMARD	Target pathway	Drug	Clinical trial evidence: study population	Assessment/ scoring type^a^	Radiographic assessment time point, week
csDMARD		Methotrexate	GO-DACT [69] Adults (≥ 18 years) with PsA (CASPAR criteria), ≥ 1 digit with tender dactylitis and ≥ 1 other site of active inflammation (joints, enthesis, spine, skin or nails), MTX-naïve, bDMARD-naïve, refractory to ≥ 2 NSAIDs at optimal dosage for 3 months.	Dactylitis total MRI score, PsAMRIS	24
			COMPLETE-PSA [70] Patients (≥ 16 years) with active PsA (≥ 2 swollen joints; dactylitis counting as 1 swollen joint).	Radiographs	No radiographs at study visits (weeks 8 & 16)
			CONTROL [71] Adults (≥ 18 years) diagnosed with PsA (CASPAR criteria) ≥ 4 weeks prior to study screening, active disease (≥ 3 swollen/≥3 tender joints), DMARD-naïve, IR^b^ to MTX (≤ 15 mg qw) for ≥ 4 weeks.	Ultrasound	4, 16 and 32
			SEAM-PsA [33] Adults (≥ 18 years) with PsA (CASPAR criteria), naïve to treatment with etanercept and other biologics, and had no prior use of MTX for PsA. Prior MTX therapy for psoriasis was allowed if discontinuation had not been due to toxicity or intolerance, and if MTX had been discontinued ≥ 6 months prior to study treatment initiation, active disease (≥ 3 swollen/≥3 tender joints), active (1 psoriatic skin lesion ≥ 2 cm diameter) plaque psoriasis.	vdH-S	24 and 48
		Leflunomide	COMPLETE-PSA [70] Patients (≥ 16 years) with active PsA (≥ 2 swollen joints; dactylitis counting as 1 swollen joint), DMARD-naïve.	Radiographs	Baseline only. No radiographs at study visits (weeks 8 & 16)
		Sulfasalazine	Department of Veterans Affairs Cooperative Study [72] Active PsA (3 joints with active arthritis, defined as joint tenderness and joint swelling of ≥ 2 on a 4-point scale and physician/patient global assessments of “moderate” on a scale of none/mild/moderate/severe/very severe) refractory to therapeutic doses of 1 of the NSAIDs, diagnosed with psoriasis.	Radiographs at screening visit	Not recorded
bDMARD	TNF	Adalimumab	ADEPT [18, 19, 60] Adults (≥ 18 years) with active PsA (≥ 3 swollen/≥3 tender or painful joints) and active psoriatic skin lesions or a documented history of psoriasis, a history of IR^b^ to or intolerance of NSAIDs for PsA.	mTSS	24
		Certolizumab pegol	RAPID-PsA [24, 25, 73] Adults (≥ 18 years) diagnosed with adult-onset PsA (CASPAR criteria) for ≥ 6 months, active disease (≥ 3 swollen/≥3 tender joints, and either ESR ≥ 28 mm/h or CRP > ULN [7.9 mg/L]), active psoriatic skin lesions or a documented history of psoriasis, refractory to ≥ 1 DMARD.	mTSS	12 and 24
		Etanercept	Etanercept vs. placebo [16, 17] Patients (18–70 years) with active PsA (≥ 3 swollen/≥3 tender joints), IR^b^ to NSAID treatment, stable plaque psoriasis with a qualifying target lesion (≥ 2 cm diameter).	mTSS	6 and 12 months
		Infliximab	IMPACT [20, 74] Adults (≥ 18 years) diagnosed with PsA for ≥ 6 months, active peripheral polyarticular arthritis (≥ 5 swollen/≥5 tender joints, ESR ≥ 28 mm/h, CRP ≥ 15 mg/L, and/or morning stiffness lasting ≥ 45 min), refractory to ≥ 1 DMARD.	vdH-S	50
			IMPACT 2 [21, 75] Adults diagnosed with PsA for ≥ 6 months, active articular disease (≥ 5 swollen/≥5 tender joints, and either CRP ≥ 15 mg/L and/or morning stiffness lasting ≥ 45 min), active (≥ 1 qualifying target lesion ≥ 2 cm diameter) plaque psoriasis, IR^b^ to current or previous DMARDs or NSAIDs, negative RF.	vdH-S	24 and 54
		Golimumab	GO-REVEAL [22, 76] Patients with active PsA (≥ 3 swollen/≥3 tender joints, negative RF, ≥ 1 subset of PsA), active (≥ 1 qualifying target lesion ≥ 2 cm diameter) plaque psoriasis, refractory to DMARDs or NSAIDs.	vdH-S	24 and 52
			GO-VIBRANT [23] Adults (≥ 18 years) diagnosed with PsA (CASPAR criteria) for ≥ 6 months, active disease (≥ 5 swollen/≥5 tender joints, CRP ≥ 0.6 mg/dL) despite current or previous DMARDs (≥ 3 months) and/or NSAIDs (≥ 4 weeks) or demonstrated intolerance for these agents.	vdH-S	24
	IL-12/23	Ustekinumab	PSUMMIT-1 and − 2 [26] Adults diagnosed with active PsA (≥ 5 swollen/≥5 tender joints, CRP ≥ 3 mg/L) for ≥ 6 months and active or documented history of plaque psoriasis, despite ≥ 3 months of DMARDs and/or ≥ 4 weeks of NSAIDs.	vdH-S	24 and 52
	IL-17 A	Ixekizumab	Ixekizumab vs. adalimumab [77] Patients diagnosed with PsA (CASPAR criteria) for ≥ 6 months, active disease (≥ 3 swollen/≥3 tender/painful joints), active plaque psoriasis affecting ≥ 3% BSA, IR^b^ to ≥ 1 csDMARD, bDMARD- and JAK-naïve.	DAPSA, PASDAS, LDI-B	24
			SPIRIT-P1 [29] Adults (≥ 18 years) diagnosed with PsA (CASPAR criteria) for ≥ 6 months, active disease (≥ 3 swollen/≥3 tender joints, and either ≥ 1 PsA-related hand or foot joint erosion on centrally read radiographs or CRP > 6 mg/L), and evidence of plaque psoriasis.	mTSS	24 and 52
		Secukinumab	EXCEED [78] Adults (≥ 18 years) with PsA (CASPAR criteria), active disease (≥ 3 swollen/≥3 tender joints), active plaque psoriasis (≥ 1 plaque ≥ 2 cm diameter, or nail changes consistent with psoriasis, or documented history of plaque psoriasis), biologic-naïve, IR^b^ to csDMARDs or had stopped csDMARDs due to safety or tolerability issues, and IR^b^ to NSAIDs for ≥ 4 weeks before study randomization.	DAPSA, PASDAS	52
			FUTURE 1 [27] Adults (≥ 18 years) with PsA (CASPAR criteria), active disease (≥ 3 swollen/≥3 tender joints) despite prior NSAIDs, DMARDs, or TNFi.	vdH-S	16 or 24, and 52
			FUTURE 5 [28] Adults (≥ 18 years) with PsA (CASPAR criteria), moderate-to-severe disease for ≥ 6 months (≥ 3 swollen/≥3 tender joints despite ≥ 4 weeks’ NSAIDs or NSAID-IR^b^), and active or documented history of plaque psoriasis or psoriatic nail changes.	vdH-mTSS	24
	IL-17 A/F	Bimekizumab	BE OPTIMAL [79] Adults (≥ 18 years) diagnosed with adult-onset PsA (CASPAR criteria) for ≥ 6 months, active disease (≥ 3 swollen/≥3 tender joints), ≥ 1 active psoriatic lesions and/or a documented history of psoriasis.	vdH-mTSS	24
			BE VITAL [80] Adults (≥ 18 years) diagnosed with adult-onset PsA (CASPAR criteria) for ≥ 6 months, and a history of IR^b^ to or intolerance of ≤ 2 TNFi for either PsA or psoriasis.	DAPSA, PASDAS	52
	IL-23-p19	Guselkumab	COSMOS [81] Adults with PsA (CASPAR criteria) and active disease (≥ 3 swollen/≥3 tender joints), active (≥ 1 psoriatic plaque ≥ 2 cm diameter) or documented history of plaque psoriasis, and a history of IR^b^ to ≤ 2 TNFi.	DAPSA, PASDAS	24
			DISCOVER-1 [82] Adults with active PsA (≥ 3 swollen/≥3 tender joints, CRP ≥ 0.3 mg/dL) despite standard therapies, a history of IR^b^ to or intolerance of standard treatment, including ≥ 4 months of non-DMARDs, or ≥ 4 weeks of NSAIDs for PsA. About 30% of study participants could have previously received one or two TNFi.	Dactylitis	24
			DISCOVER-2 [83] Biologic-naïve adults with active PsA (≥ 5 swollen/≥5 tender joints), CRP ≥ 0.6 mg/dL, current or documented history of psoriasis, and either IR^b^ to or intolerance of standard non-biologic therapy (e.g., csDMARDs, NSAIDs and/or apremilast).	vdH-S	24, 52 and 100
		Risankizumab	KEEPsAKE 2 [84] Adults with active PsA (≥ 5 swollen/≥5 tender joints), active (≥ 1 psoriatic plaque ≥ 2 cm diameter) plaque psoriasis, biologic-IR^c^ and/or csDMARD-IR^d^.	DAPSA, LDI	24
	CTLA4	Abatacept	ASTRAEA [30] Adults (≥ 18 years) with PsA (CASPAR criteria), active disease (≥ 3 swollen/≥3 tender joints), active (≥ 1 psoriatic plaque ≥ 2 cm diameter) plaque psoriasis, and IR^b^ to or intolerance of ≥ 1 non-bDMARD.	vdH-S	24 and 52
tsDMARD	PDE4	Apremilast	Real-world evidence [85] Adults (≥ 18 years) with PsA (CASPAR criteria) who had initiated apremilast during the previous 6 (± 1) months and had not previously received biologic treatment.	Not defined	6 and 12 months
			MOSAIC [86] Adults (≥ 18 years) with active PsA (≥ 3 swollen/≥3 tender joints, and hand involvement of ≥ 1 swollen joint or dactylitis according to SPARCC or LEI criteria).	cDAPSA, hand and whole-body MRI, PsAMRIS, OMERACT PsAMRIS, OMERACT MRI-WIPE, SPARCC enthesitis index	24 and 48
	JAK	Tofacitinib	Phase 3 study [31] Adults (≥ 18 years) diagnosed with PsA (CASPAR criteria) for ≥ 6 months, active disease (≥ 3 swollen/≥3 tender/painful joints), active plaque psoriasis, IR^b^ to ≥ 1 csDMARD, TNFi-naïve.	vdHmTSS	12 months
			OPAL Broaden [32] Adults (≥ 18 years) diagnosed with PsA (CASPAR criteria) for ≥ 6 months, IR^b^ to ≥ 1 csbDMARD, TNFi-naïve.	vdH-S	12 months
		Upadacitinib	SELECT-PsA 1 [87] Adults (≥ 18 years) with active PsA (CASPAR criteria) and IR^b^ to or intolerance of ≥ 1 non-bDMARD.	mTSS	104 (2 years)

^**a**^Radiographic scoring methods were used as well as surrogate markers, consisting of the Dactylitis total MRI score, the DAPSA, cDAPSA, LDI, LDI-B, and PASDAS indices; ^b^Inadequate response (IR) was characterized as lack of efficacy or intolerance; ^c^Biologic-IR was characterized as a demonstrated lack of efficacy after ≥ 12 weeks of treatment, or intolerance to one or two eligible biologic agents; ^d^csDMARD-IR was characterized as a demonstrated lack of efficacy after ≥ 12 weeks or intolerance or a contraindication to methotrexate, sulfasalazine, leflunomide, apremilast, bucillamine, iguratimod or ciclosporin A. bDMARD, biologic disease-modifying antirheumatic drug; BSA, body surface area; CASPAR, ClASsification for Psoriatic Arthritis; cDAPSA, Clinical Disease Activity Index for Psoriatic Arthritis; CRP, C-reactive protein; csDMARD, conventional synthetic disease-modifying antirheumatic drug; CTLA4, cytotoxic T lymphocyte antigen 4; DAPSA, Disease Activity Index for Psoriatic Arthritis; DMARD, disease-modifying antirheumatic drug; DSS, Dactylitis Severity Score; ESR, erythrocyte sedimentation rate; IL, interleukin; IR, inadequate response; JAK, Janus kinase; LDI, Leeds Dactylitis Index; LDI-B, Leeds Dactylitis Index–Basic; LEI, Leeds Enthesitis Index; min, minutes; MOSAIC, *MRI* magnetic resonance imaging, *MRI-WIPE* MRI Whole-Body Score for Inflammation in Peripheral Joints and Entheses in Inflammatory Arthritis, *mTSS* van der Heijde modified Total Sharp Score, *MTX* methotrexate, *NSAID* nonsteroidal anti-inflammatory drug, *OMERACT* Outcome Measures in Rheumatology Clinical Trial, *PASDAS* Psoriatic Arthritis Disease Activity Score, *PDE4* phosphodiesterase 4, *PsA* psoriatic arthritis, *PsAMRIS* psoriatic arthritis MRI score, *qw* every week, *RF* rheumatoid factor, *Sharp* PsA-modified Sharp method, *SPARCC* Spondyloarthritis Research Consortium of Canada, *TNF* tumor necrosis factor, *TNFi* tumor necrosis factor inhibitor, *tsDMARD* targeted synthetic disease-modifying antirheumatic drug, *ULN* upper limit of normal, *vdH-S* PsA-modified van der Heijde-Sharp score, *vdHmTSS* van der Heijde modified Total Sharp Score

### International guidelines on PsA treatment algorithms

Although the 2021 GRAPPA recommendations and the 2023 EULAR recommendations both strongly recommend the use of csDMARDs such as methotrexate, leflunomide, and sulfasalazine as first-line therapy for PsA, they lack a specific order for using different types of DMARDs [37, 90]. It is important to note that these agents do not effectively inhibit RP and new, high-quality data support the use of bDMARD treatment using TNF inhibitors, IL-17 inhibitors, IL-23 inhibitors, and JAK inhibitors, in preference to csDMARDs as first-line therapy, especially in early PsA; moderate-quality evidence supports IL-12/23 inhibitors or PDE4 inhibitors over placebo [90].

#### TNF inhibition

TNF inhibitors are highly effective in the treatment of PsA, with significant improvements in articular manifestations of the disease and proven efficacy in the inhibition of radiographic joint damage [17, 18, 75]. In the ADEPT study, adalimumab treatment reduced the risk of RP in patients with PsA by approximately 5-fold and was of most radiographic benefit in patients with the greatest CRP concentrations at baseline [62]. However, not all patients with PsA respond to TNF inhibitors and therefore need additional treatment modalities that have distinct mechanisms of action. Furthermore, other PsA cases become refractory to TNF inhibitors after a certain period of use, or PsA may be recurrent or persist despite TNF inhibitor treatment [91].

#### Inhibition of the interleukin pathway in PsA

Clinical trial data have shown that using ustekinumab, a fully human immunoglobulin G1κ (IgG1κ) monoclonal antibody against IL-12p40, the common subunit of IL-12 and IL-23, effectively inhibits a different inflammatory aspect of PsA, and has demonstrated good safety at 6 months and 1 year [26, 92]. In addition, IL-17 inhibitors suppress RP in PsA [93]. The IL-23 inhibitors risankizumab and guselkumab have also shown efficacy in reducing the rates of RP in patients with PsA, including patients with active PsA who have previously had inadequate response or were intolerant to one or more biological therapies or one or more csDMARDs [83, 94, 95]. In particular, in active PsA, guselkumab has shown that blockade of the IL-23 pathway may modify long-term PsA disease signs and symptoms, and prevent further joint damage, with significantly less structural joint damage observed at 2 years by those patients who experienced greater improvement in DAPSA scores after 8 weeks of guselkumab treatment [96].

The IL-17 A inhibitors secukinumab and ixekizumab are also associated with lower rates of RP in PsA [27, 97–100], with the FUTURE-5 study showing that secukinumab effectively inhibited radiographic structural progression through 2 years in patients with PsA [98]. Similarly, the monoclonal IgG1 antibody bimekizumab, a selective IL-17 F/A inhibitor, was associated with superior improvements compared with placebo in the inhibition of RP after 16 and 52 weeks of treatment in the phase 3 BE OPTIMAL trial in patients with PsA who were naïve to bDMARDs [79, 101]. Bimekizumab showed sustained efficacy after 52 weeks of treatment in the open-label extension BE VITAL of the BE COMPLETE study, which involved patients with active PsA and prior inadequate response to TNF inhibitors [80].

The JAK inhibitors tofacitinib and upadacitinib have shown long-term efficacy at maintaining inhibition of RP in clinical trials involving patients with PsA [87]. In one trial, upadacitinib 15 mg or 30 mg was more effective than adalimumab 15 mg or 30 mg at inhibiting RP through week 104 [87]. Interestingly, in the OPAL Broaden phase 3 trial, which enrolled patients with active PsA with a prior inadequate response to csDMARDs, daily tofacitinib 10 mg or 20 mg was not superior to adalimumab 40 mg once every 2 weeks in the inhibition of RP assessed after 12 months [32]. Changes from baseline in radiographic outcomes were minimal and similar across all three treatment groups [32].

Evidence from a network meta-analysis examining the effects of US Food and Drug Administration (FDA)-approved bDMARD regimens found that most are more effective than placebo at inhibiting RP in PsA, and that some bDMARDs are superior to others in preventing RP [5]. It has been acknowledged that clinicians face significant challenges when seeking to achieve a good outcome and inhibit RP in patients who present with PsA [2].

## Conclusions

While there are unmet needs to clarify and define RP, this review will assist rheumatologists to assess the extent of structural damage in primarily the peripheral forms of PsA in clinical practice. We found that the SvdH RA scoring method was employed most frequently to determine radiographic scores. Taking into account that the factors that lead to a more aggressive, faster, or more active RP are related to the number of activity indicators (overweight, smoking, etc.) and since high psoriatic activity correlates with greater progression, the information provided in the present review would suggest that in certain types of patients with a specific risk factor profile (e.g. smokers, overweight, etc.) and increased disease progression, within the context of clinical trials, the assessment of structural damage by measuring RP at 6 months of follow-up might already be sufficient to determine structural damage. This would facilitate the development of these trials and future clinical research.

Importantly, it is necessary to continue researching and investing in real-world evidence studies, and also prospective observational studies including cohorts of real-life patients, so that the observations reviewed in the current article can be translated into routine clinical practice. New, highly sensitive techniques are increasingly being used to determine the extent of PsA-related structural damage, such as magnetic resonance imaging and ultrasound scans. Researchers have expressed a keen interest in continuing to work with these new measurement methods that can support current radiographic determinations, and accurately assess RP.

## Data Availability

Data availability is not applicable to this article as no new data were generated or analyzed in this narrative review.

## Abbreviations

ADEPT: Adalimumab Effectiveness in PsA Trial
ASAS: Assessment of SpondyloArthritis international Society
axSpA: Axial spondyloarthritis
bDMARD: Biological disease-modifying antirheumatic drug
CCP: Cyclic Citrullinated peptide
CPDAI: Composite Psoriatic Disease Activity Index
CRP: C-reactive protein
csDMARD: Conventional synthetic disease-modifying antirheumatic drug
CTLA4: Cytotoxic T-lymphocyte-associated antigen 4
DAPSA: Disease Activity index for PsA
DAS28: 28-joint Disease Activity Score
EULAR: European League Against Rheumatism
GRACE: Group for Research and Assessment of Psoriasis and Psoriatic Arthritis Composite Exercise
ICAM-1: Intercellular adhesion molecule-1
IgG1κ: Immunoglobulin G1κ
IL: Interleukin
JAK: Janus kinase
MDA: Minimal disease activity
MIP-1β: Macrophage inflammatory protein-1 beta
MTX: Methotrexate
PsAM: Psoriatic arthritis mutilans
PARS: Psoriatic arthritis Ratingen score
PASDAS: Psoriatic Arthritis Disease Activity Score
PDE4: Phosphodiesterase-4
PsA: Psoriatic arthritis
RA: Rheumatoid arthritis
RP: Radiographic progression
SAA: Serum amyloid A
SASSSm: Modified Stoke Ankylosing Spondylitis Spine Score
SvdH: Sharp-van der Heijde
T2T: Treat-to-target
TNF: Tumor necrosis factor
tsDMARD: Targeted synthetic disease-modifying antirheumatic drug
